# The Burden of Malnutrition and Fatal COVID-19: A Global Burden of Disease Analysis

**DOI:** 10.3389/fnut.2020.619850

**Published:** 2021-01-21

**Authors:** Elly Mertens, José L. Peñalvo

**Affiliations:** Unit of Noncommunicable Diseases, Department of Public Health, Institute of Tropical Medicine, Antwerp, Belgium

**Keywords:** malnutrition, undernutrition, overnutrition, BMI, global burden, COVID-19 mortality

## Abstract

**Background:** Although reasonable to assume, it is not yet clear whether malnourished countries are at higher risk for severe or fatal coronavirus disease 2019 (COVID-19). This study aims to identify the countries where prevalent malnutrition may be a driving factor for fatal disease after severe acute respiratory syndrome coronavirus 2 (SARS-CoV-2) infection.

**Methods:** Using estimates from the Global Burden of Disease 2019, country-level burden of malnutrition was quantified using four indicators: death rates for child growth failure (underweight, stunting, and/or wasting) and years lived with disability (YLD) attributed to iron and vitamin A deficiencies and high body mass index (BMI). Global mortality descriptors of the ongoing COVID-19 pandemic were extracted from the European Centre for Disease Prevention and Control, and case fatality ratios (CFRs) were calculated introducing a lag time of 10 weeks after the first death of a confirmed case. Bivariate analyses for 172 countries were carried out for malnutrition indicators and fatal COVID-19. Correlations between burden indicators were characterized by Spearman's rank correlation coefficients (ρ) and visually by scatterplots. Restricted cubic splines and underlying negative binomial regressions adjusted for countries' age-structure, prevalent chronic comorbidities related to COVID-19, population density, and income group were used to explore non-linear relationships.

**Results:** Stratified by the World Bank income group, a moderate positive association between YLD rates for iron deficiency and CFRs for COVID-19 was observed for low-income countries (ρ = 0.60, *p* = 0.027), whereas no clear indications for the association with child growth failure, vitamin A deficiency, or high BMI were found (ρ < 0.30). Countries ranking high on at least three malnutrition indicators and presenting also an elevated CFR for COVID-19 are sub-Saharan African countries, namely, Angola, Burkina Faso, Chad, Liberia, Mali, Niger, Sudan, and Tanzania, as well as Yemen and Guyana.

**Conclusions:** Population-level malnutrition appears to be related to increased rates of fatal COVID-19 in areas with an elevated burden of undernutrition, such as countries in the Sahel strip. COVID-19 response plans in malnourished countries, vulnerable to fatal COVID-19, should incorporate food security, nutrition, and social protection as a priority component in order to reduce COVID-19 fatality.

## Introduction

The ongoing pandemic of the coronavirus disease 2019 (COVID-19), caused by severe acute respiratory syndrome coronavirus 2 (SARS-CoV-2), is a major cause of morbidity and mortality in 2020 across the globe, affecting more than 200 countries. Internationally, countries have reacted to COVID-19 by introducing key public health non-pharmaceutical practices, such as handwashing (1), physical distancing (2), and wearing facemasks (3) to hinder the spread of the virus and to protect vulnerable populations. Evidence, mainly from high-income countries (HIC), has emerged suggesting that the risk of suffering severe COVID-19 outcomes is higher in elderly populations and in individuals with pre-existing chronic comorbidities (4–6). Older age and multi-morbidity are also often associated with a higher risk and prevalence of disease-related malnutrition (7–9). It has been postulated that the nutritional status might also play a key role as a driver of SARS-CoV-2 virulence (10, 11). In this line, current patient care recommendations, as those published by the European Society for Clinical Nutrition and Metabolism (ESPEN) (12), among others (13, 14), include nutritional interventions for critical patients suffering from the SARS-CoV-2 infection.

Overall, malnutrition is the primary cause of immunodeficiency worldwide, affecting mostly infants, children, adolescents, and the elderly, increasing their vulnerability to infections (15). Nutritional status might be of particular concern in low- and lower–middle-income countries facing the so-called double burden of malnutrition, i.e. the coexistence of childhood undernutrition (commonly characterized by growth failure and/or micronutrient deficiencies) and overnutrition (overweight/obesity), affecting all levels of the population (countries, communities, families, and individuals) (16). Available evidence from HIC indicates that overweight and obesity as a form of malnutrition is a key predictor for severe COVID-19 at the individual level, and this also holds for young adults with no underlying conditions (17, 18). It is, however, reasonable to assume that also undernourished populations, including survivors of undernourishment, who often develop weaker immune systems, may be at greater risk of severe or fatal COVID-19 illness, since childhood malnutrition is associated with high morbidity and mortality globally, mainly due to infectious diseases (19).

To date, it is not yet clear whether population-level nutritional status plays a role in the population-level vulnerability to COVID-19. The aim of this study was therefore to identify the countries where a high burden of malnutrition coincides with higher rates of fatal disease after SARS-CoV-2 infection, indicating a potential relationship between these burdens.

## Materials and Methods

### Data Sources

Country-level publicly available information for both exposure and outcome variables was used for these analyses. Indicators of countries' burden of malnutrition, defined as child growth failure, iron deficiency, vitamin A deficiency, and high body mass index (BMI), were obtained from the Global Burden of Disease (GBD) Study 2019 (20, 21). Child growth failure, including child underweight, child wasting, and child stunting, is a common indicator of malnutrition among children under 5 years old (22), whereas iron deficiency is the most widespread micronutrient deficiency worldwide affecting all layers of the population (23), followed by vitamin A deficiency, also especially burdensome in children under five (24). High BMI, defined as BMI ≥25 kg/m^2^ for adults (aged 20+ years) and using thresholds from the International Obesity Task Force standards for children (aged <20 years), was used as a measure of countries' burden of malnutrition due to overnutrition. The GBD global health data exchange (GHDx) tool (25) was used to extract rates of deaths for child growth failure and rates of years lived with disability (YLD) for iron and vitamin A deficiencies and high BMI for both sexes and all ages in the year 2019, for each country. Rates of deaths for child growth failure (per 100,000) provide an understanding to the severity of underweight, wasting, or stunting to fatal outcomes at the country level. Rates of YLD (per 100,000) for iron and vitamin A deficiencies and high BMI are defined as the disability-adjusted prevalence of the conditions and reflect the non-fatal burden of malnutrition, and thus adding further insight to the severity of living with iron and/or vitamin A deficiency and/or high BMI at the country level.

To account for other factors related to both malnutrition and COVID-19, we adjusted our models with YLD rates for COVID-19-related vulnerable health conditions, as identified by WHO (26), as well as the percentage of the population aged 65 and older, population density, and income group. The YLD rate for COVID-19-related vulnerable health conditions was calculated for each country as the sum of YLD rates for chronic respiratory diseases, chronic heart diseases, chronic kidney diseases, chronic liver diseases, chronic neurological conditions, diabetes, problems with the spleen (e.g., sickle cell disorders, sickle cell trait), and cancers, as extracted from the GHDx tool for both sexes and all ages in the year 2019 (25). The percentage of the population aged 65 years and older was extracted from the World Bank (27), as well as the population density (28) and the countries' income classification, available for the year 2019 for 218 countries (29).

Outcome variables were obtained from Our World in Data (30), as reported by the European Centre for Disease Prevention and Control (ECDC), including total cases and total deaths (absolute and per million), available for 209 countries. From these data, we calculated weekly case fatality ratios (CFRs) for each country as the ratio between confirmed deaths and confirmed cases using the average of CFR for each day of that week starting from the day following the first death reported. Accounting for the potential biases introduced by calculating CFR during the early phases of the outbreak and to enable comparison between countries facing different stages of the pandemic, the present analyses included only countries with information on COVID-19 mortality at week 10 following the first death reported of a confirmed case and using CFR at that time point as a measure for countries' vulnerability to fatal COVID-19 (31).

### Analyses

The present analyses included 172 countries for which data were available on both countries' burden of malnutrition and COVID-19 CFR. Each measure was divided in tertiles (low, mid, high) to facilitate visualization. Countries falling in the higher tertile of each measure were classified as highly vulnerable to either malnutrition or COVID-19 mortality. Bivariate groupings were established by considering the overlapping of the tertiles for measures of malnutrition and COVID-19. Relationships were visualized in scatterplots with the burden of malnutrition on the X-axis and CFRs on the Y-axis and using colors to depict income groups. Potential non-linear relationships at the global level were described using restricted cubic splines (RCS) with knots placed at the 5th, 35th, 65th, and 95th percentiles (32). The underlying negative binomial models for the RCS analyses used CFR for COVID-19 at week 10 following the first death reported as dependent variable and the measures of a countries' burden of malnutrition (child growth failure, iron deficiency, vitamin A deficiency, and high BMI) as independent variables. All models were adjusted as described above. Spearman's rank correlation coefficients (ρ) were used to describe the correlation between malnutrition measures and the CFR for COVID-19 at the global level and at the income group level (29), and *p*-values were adjusted for multiple testing according to Sidak. In addition, at the global level and stratified by income group, negative binomial models were used to compute adjusted prevalence ratios (PRs) and their 95% confidence intervals (CIs) for the relationship between a countries' burden of malnutrition and COVID-19 fatality, with countries classified into tertiles depending on their burden of malnutrition, using the lowest tertile (lowest burden) as reference.

Using the countries' position in the scatterplot, a heatmap of the world was created to visualize the countries where malnutrition is likely to be a driver for fatal COVID-19. Malnourished countries vulnerable to fatal COVID-19 were those countries with high rates (third tertile) for at least three malnutrition measures and a high (third tertile) CFR.

Sensitivity analyses included using the calculation of CFRs at week 15 following the first COVID-19 death reported, and the CFRs measured on the most recent day of the pandemic at the time of analyses (04/10/2020). As a measure of COVID-19 severity, the cumulative number of the intensive care unit (ICU) beds needed until week 10 following the first report of a confirmed COVID-19 death, as extracted from the GBD (25), was used in a secondary analysis to test the robustness of the results. A two-sided *p*-value below 0.05 was considered as statistically significant, and all analyses were carried out using STATA (Release 16/SE; StataCorp LP, College Station, TX, USA).

## Results

### Countries' Burden of Malnutrition

Supplementary Table 1 shows for each country its vulnerability to fatal COVID-19 due to malnutrition, ordered by income group and CFR. Countries' burden of undernutrition decreased by income group, as measured by child growth failure and iron and vitamin A deficiencies. Median death rates for child growth failure per 100,000 assessed for both sexes and all ages were 45.5 (IQR 34.5, 85.2) in low-income countries (LIC), followed by 12.5 (IQR 5.6, 28.8) in lower–middle- and 1.86 (IQR 0.89, 5.81) in upper–middle-income countries, and were the lowest in HIC with a median rate of 0.62 (IQR 0.25, 1.76). Median YLD rates for iron deficiencies were 679 (IQR 504, 797) in LIC, followed by 435 (IQR 239, 595) in lower–middle- and 191 (IQR 137, 285) in upper–middle-income countries, and were the lowest in HIC with a median rate of 85 (IQR 33, 139). Median YLD rates for vitamin A deficiencies were 61.3 (IQR 40.1 86.6) in LIC, followed by 20.3 (IQR 9.54, 37.4) in lower–middle- and 5.03 (IQR 2.25, 8.71) in upper–middle-income countries, and were the lowest in HIC with a median rate of 5.03 (IQR 0.12, 1.16).

When countries' burden of overnutrition was assessed, the rates increased by income group. Median YLD rates for high BMI were 161 (IQR 132, 210) in LIC, followed by 351 (IQR 253, 509) in lower–middle- and 779 (IQR 622, 987) in upper–middle-income countries, and were the highest in HIC with a rate of 947 (IQR 736, 1,145).

### Countries' Burden of COVID-19

Total COVID-19 confirmed cases at week 10 following the first death reported per 100,000 were the highest in HIC (1,516 cases; IQR 467, 3,138), followed by upper–middle- (318; IQR 133, 1,223) and lower–middle-income countries (161; IQR 64, 441), and were the lowest in LIC (72; IQR 29, 288). In addition, total COVID-19 deaths were the highest in HIC (32 deaths; IQR 15, 96), followed by upper–middle- (9.3; IQR 2.6, 37.8) and lower–middle-income countries (3.91; IQR 1.22, 8.05), and were the lowest in LIC (2.4; IQR 0.40, 5.50). Countries' vulnerability to fatal COVID-19, as measured by CFR, was the highest in HIC (3.9; IQR 1.7, 6.9), followed by lower–middle- (2.8; IQR 1.1, 3.9) and upper–middle-income countries (2.6; IQR 1.3, 4.8), and the lowest in LIC (1.8; IQR 0.9, 4.1).

### Are Malnourished Countries More Vulnerable to Fatal COVID-19?

The potential relationship between prevalent malnutrition and COVID-19 fatality globally was assessed by each malnutrition indicator individually using models accounting for other recognized determinants of vulnerability to severe or fatal COVID-19 (Supplementary Figure 1), in an attempt to isolate the potential association of malnutrition with fatal COVID-19 (26); in particular for HIC only, a moderate positive correlation of CFR for COVID-19 with YLD rates for COVID-19 vulnerable health conditions (ρ = 0.58, *p* < 0.001) and with the percentage of individuals aged 65 or above (ρ = 0.49, *p* = 0.006) was observed.

**Figure 1:**
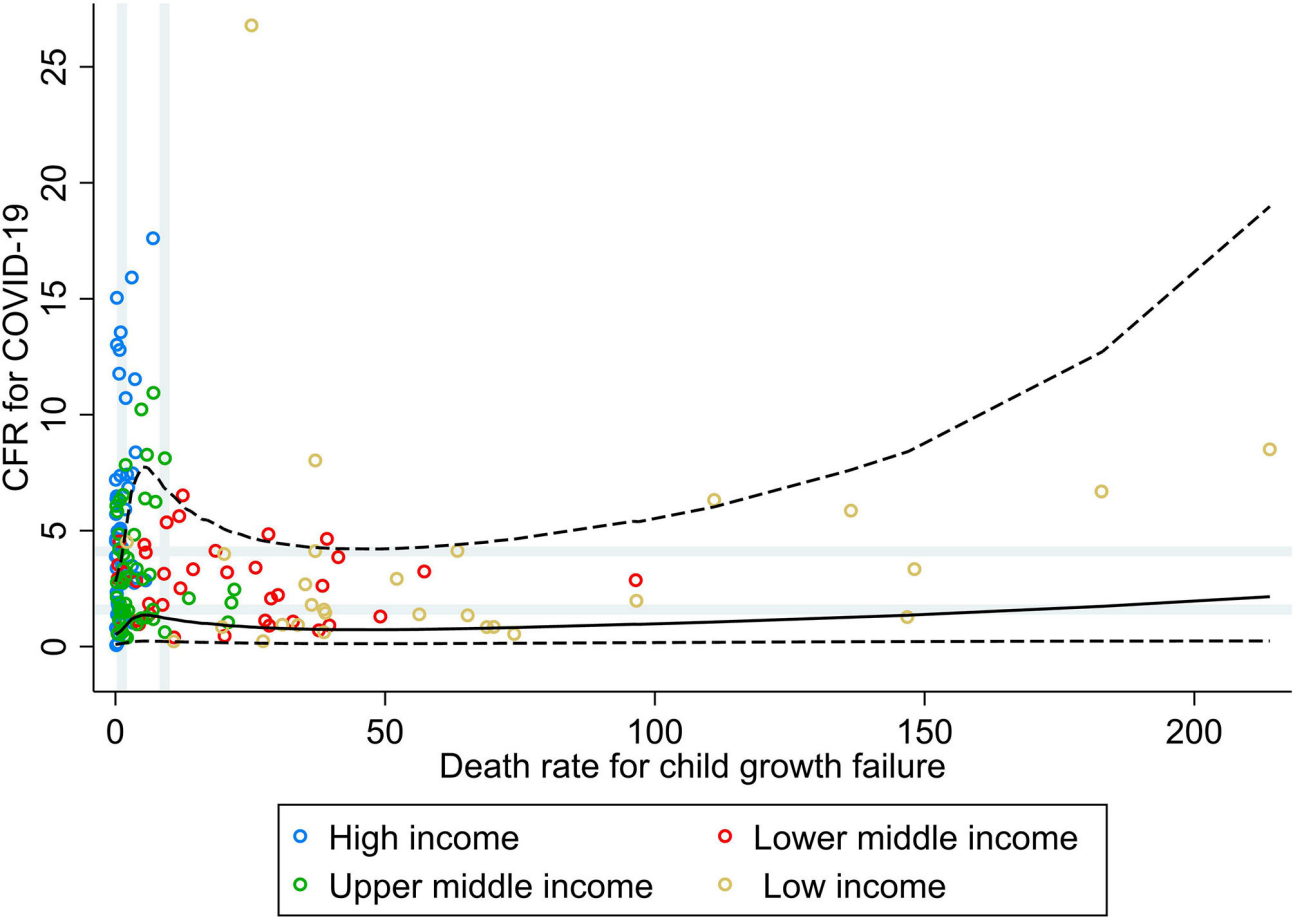
Scatterplot of death rate for child growth failure (underweight, wasting, or stunting) against the average case fatality ratio for COVID-19 at week 10 following the first confirmed death reported, stratified by income group (29). All rates described are crude rates per 100,000 population. Gray vertical and horizontal gridlines indicate tertiles dividing lines for the measures: death rates for child growth failure (at 1.2 and 9.1) and case fatality ratio (CFR) for COVID-19 (at 1.6 and 4.1). Solid black line represents the restricted cubic spline, showing the shape of the adjusted association on a continuous scale with knots at the 5th, 35th, 65th, and 95th percentiles (corresponding to death rate for child growth failure of 0.2, 1.3, 7.4, and 74, respectively), with dashed black lines indicating the 95% confidence intervals.

Figure 1 shows the bivariate analyses stratified by income group and the non-linear shape of the adjusted association between rate of death for child growth failure and CFR for COVID-19, obtained by RCS analysis. No correlation was seen between the rate of death for child growth failure and CFR for COVID-19 at the global level, nor at the income group level (ρ < 0.30; Table 1). Countries' vulnerability to fatal COVID-19 seemed to be higher across increasing rates for child growth failure (PR 1.76; 95% CI 0.92, 3.37), mainly driven by the association seen in upper–middle-income countries.

**Table 1:** Spearman's rank correlation and prevalence ratio for countries' burden of malnutrition and fatal COVID-19, measured using CFR at week 10 following the first confirmed death reported, stratified by income group^*^.

	**Death rate for child growth failure**	**YLD rate**		
		**Iron deficiency**	**Vitamin A deficiency**	**High BMI**
**Global (*****n*** **=** **172)**				
Tertile 1, ref	<1.2	<139	<1.5	<441
Tertile 2	1.2–9.1	139–349	1.5–12	441–833
Tertile 3	≥9.1	≥349	≥12	≥833
ρ (*p*-value)	−0.09 (1.000)	−0.21 (0.186)	−0.16 (0.796)	0.26 (0.018)
PR, T3 vs. T1 (95% CI)	1.76 (0.92; 3.37)	0.87 (0.47; 1.61)	1.58 (0.70; 3.53)	1.77 (0.77; 4.09)
**High-income countries (*****n*** **=** **57)**				
Tertile 1, ref	<0.4	<43	<0.1	<781
Tertile 2	0.4–1.1	43–124	0.1–0.6	781–1,071
Tertile 3	≥1.1	≥124	≥0.6	≥1,071
ρ (*p*-value)	0.26 (0.815)	−0.15 (1.000)	−0.07 (1.000)	0.27 (0.775)
PR, T3 vs T1 (95% CI)	1.36 (0.76; 2.43)	0.74 (0.32; 1.71)	1.67 (0.82; 3.39)	0.79 (0.32; 1.98)
**Upper–middle-income countries (*****n*** **=** **48)**				
Tertile 1, ref	<1.4	<152	<3.3	<663
Tertile 2	1.4–4.8	152–247	3.3–7.7	663–913
Tertile 3	≥4.8	≥247	≥7.7	≥913
ρ (*p*-value)	0.03 (1.000)	−0.23 (0.969)	0.04 (1.000)	0.22 (0.985)
PR, T3 vs T1 (95% CI)	2.08 (1.01; 4.29)	0.45 (0.25; 0.82)	0.96 (0.43; 2.12)	0.84 (0.37; 1.84)
**Lower–middle-income countries (*****n*** **=** **38)**				
Tertile 1, ref	<8.7	<294	<12	<271
Tertile 2	8.7–28	294–539	12–31	271–440
Tertile 3	≥28	≥539	≥31	≥440
ρ (*p*-value)	−0.09 (1.000)	−0.44 (0.139)	−0.14 (1.000)	0.18 (1.000)
PR, T3 vs T1 (95% CI)	1.70 (0.58; 4.99)	0.28 (0.13; 0.63)	0.80 (0.29; 2.17)	0.45 (0.17; 1.16)
**Low-income countries (*****n*** **=** **29)**				
Tertile 1, ref	<36	<526	<51	<151
Tertile 2	36–69	526–712	51–84	151–196
Tertile 3	≥69	≥712	≥84	≥196
ρ (*p*-value)	0.30 (0.979)	0.60 (0.027)	0.47 (0.333)	−0.13 (1.000)
PR, T3 vs T1 (95% CI)	1.00 (0.32; 3.19)	3.61 (1.45; 8.99)	0.83 (0.25; 2.79)	3.32 (0.66; 16.8)

* *According to World Bank 2019 (29). BMI, body mass index; CFR, case fatality ratio; CI, confidence interval; Death rate, rate of death per 100,000 for all ages; PR, prevalence ratio for COVID-19 fatality in the highest tertile of a countries' burden of malnutrition relative to the lowest tertile calculated in a negative binomial model; ρ, Spearman's rank correlation coefficient with p-value adjusted for multiple testing according to Sidak; YLD rate, rate of years lived with disability per 100,000 for all ages*.

**Figure 2:**
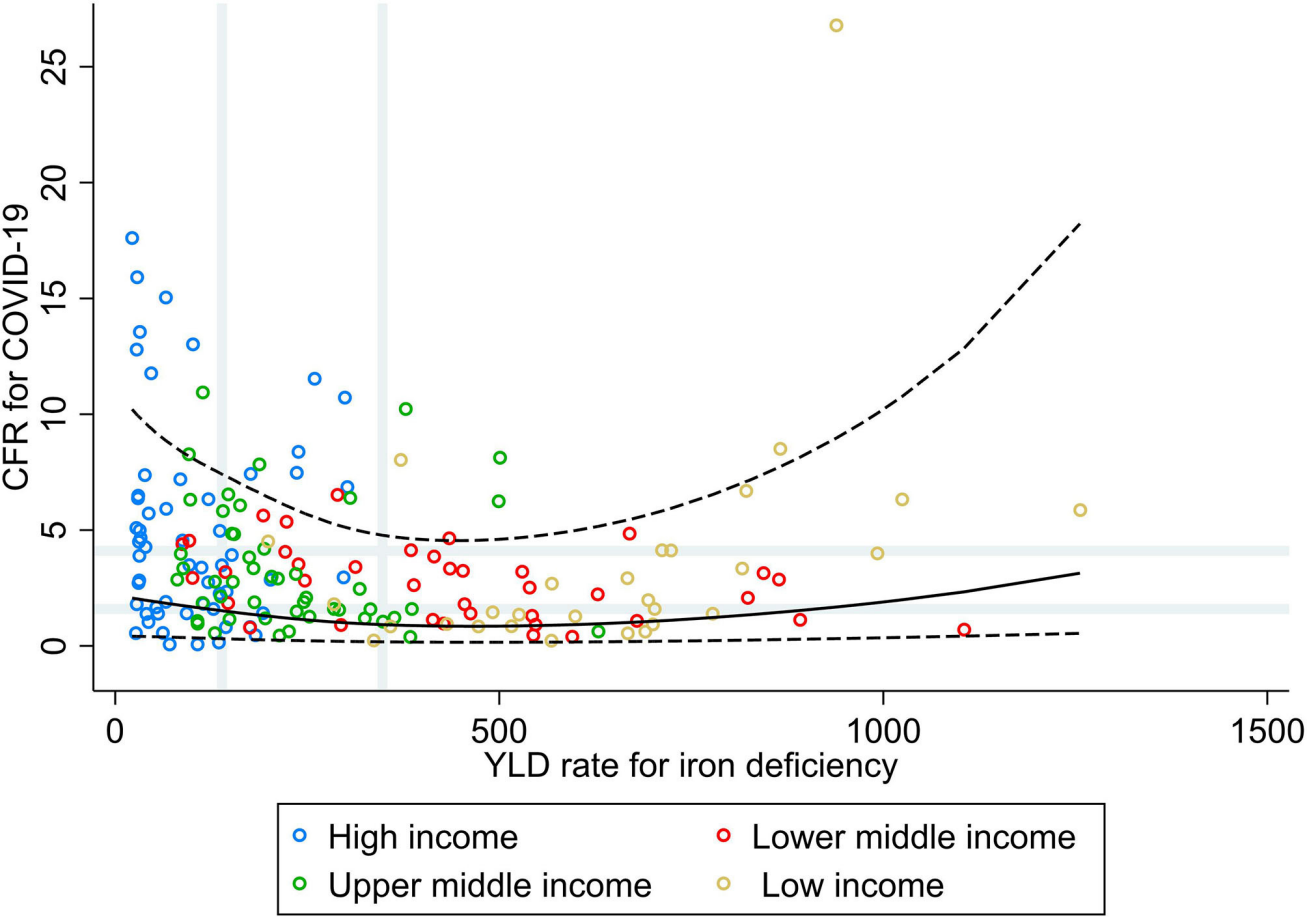
Scatterplot of years lived with disability rate for iron deficiencies against the average case fatality ratio for COVID-19 at week 10 following the first confirmed death reported, stratified by income group (29). All rates described are crude rates per 100,000 population. Gray vertical and horizontal gridlines indicate tertiles dividing lines for the measures: years lived with disability (YLD) rates for iron deficiency (at 139 and 348) and case fatality ratio (CFR) for COVID-19 (at 1.6 and 4.1). Solid black line represents the restricted cubic spline, showing the shape of the adjusted association on a continuous scale with knots at the 5th, 35th, 65th, and 95th percentiles (corresponding to YLD rate for iron deficiency of 30, 144, 325, and 844, respectively), with black dashed lines indicating the 95% confidence intervals.

Figure 2 shows that countries with low and median rates of YLD for iron deficiencies did not necessarily have the higher CFR for COVID-19, whereas a slightly higher CFR for COVID-19 was seen for the countries with very high rates of YLD for iron deficiencies. Stratified by income group, a moderate positive correlation was observed in LIC (ρ = 0.60, *p* = 0.027; Table 1), whereas in the other income groups, a weak negative correlation was observed. In addition, when comparing countries in the highest tertile for the burden of iron deficiency with the lowest, increasing rates were associated with a higher vulnerability to fatal COVID-19 in LIC only (PR 3.61; 95% CI 1.45, 8.99), whereas in the other income groups, this association was inversed and significantly inversed in lower–middle- (PR 0.28; 95% CI 0.13, 0.63) and upper–middle-income countries (PR 0.45; 95% CI 0.25, 0.82).

**Figure 3:**
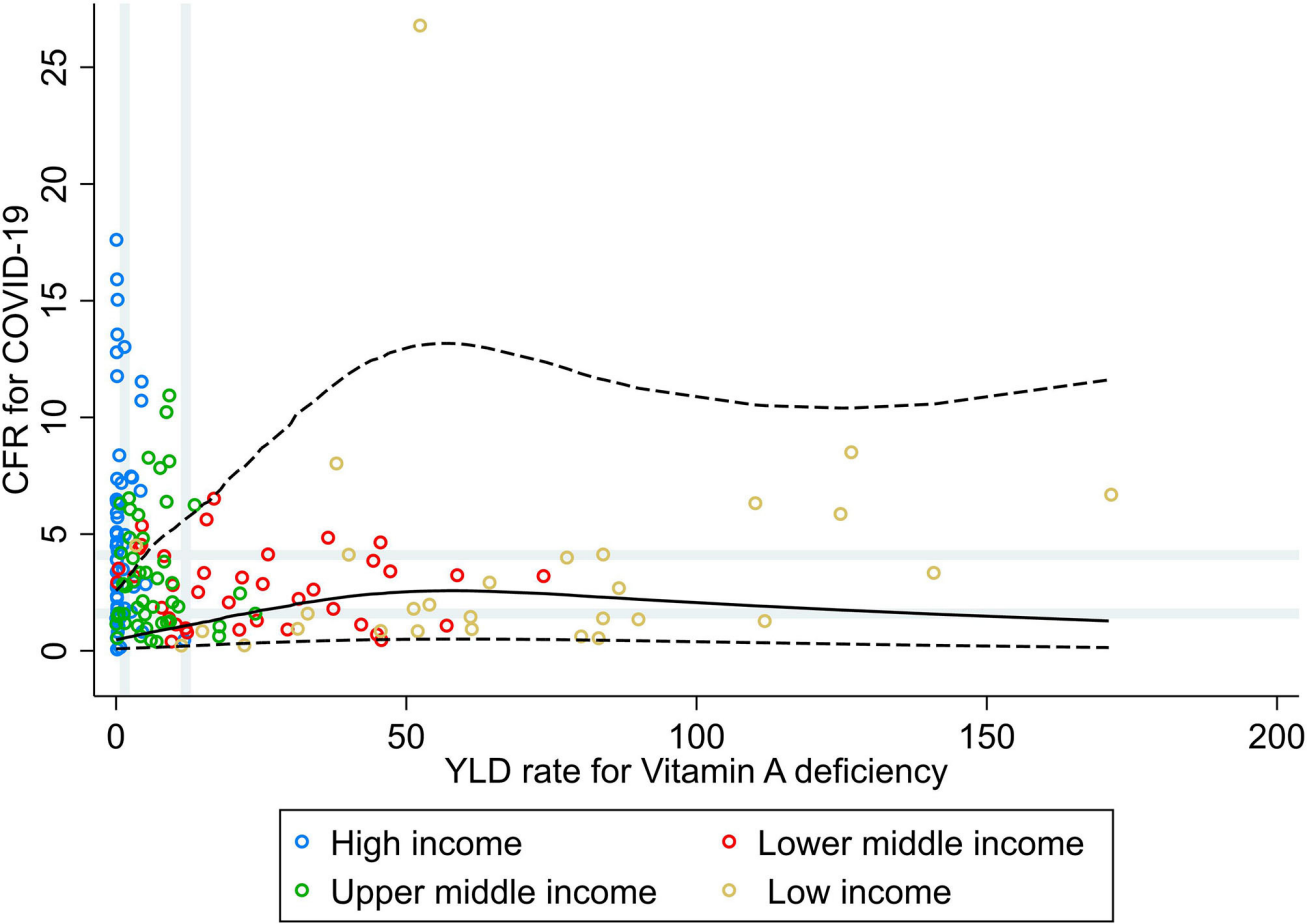
Scatterplot of years lived with disability rate for vitamin A deficiencies against the average case fatality ratio for COVID-19 at week 10 following the first confirmed death reported, stratified by income group (27). All rates described are crude rates per 100,000 population. Gray vertical and horizontal gridlines indicate tertiles dividing lines for the measures: years lived with disability (YLD) rates for vitamin A deficiency (at 1.5 and 12) and case fatality ratio (CFR) for COVID-19 (at 1.6 and 4.1). Solid black line represents the restricted cubic spline, showing the shape of the adjusted association on a continuous scale with knots at the 5th, 35th, 65th, and 95th percentiles (corresponding YLD rate for vitamin A deficiency of 0.1, 2.3, 10.7, and 84, respectively), with black dashed lines indicating the 95% confidence intervals.

Results from the RCS analysis and the bivariate analyses of YLD rate for vitamin A deficiency and CFR for COVID-19 (Figure 3) show that countries' vulnerability to fatal COVID-19 was slightly higher with increasing rates of vitamin A deficiency, with no further increases in CFR for COVID-19 for countries with very high rates of vitamin A deficiency. No correlations or associations were observed between the rate of YLD for vitamin A deficiencies and CFR for COVID-19 at the global level, nor at the income group level (Table 1).

**Figure 4:**
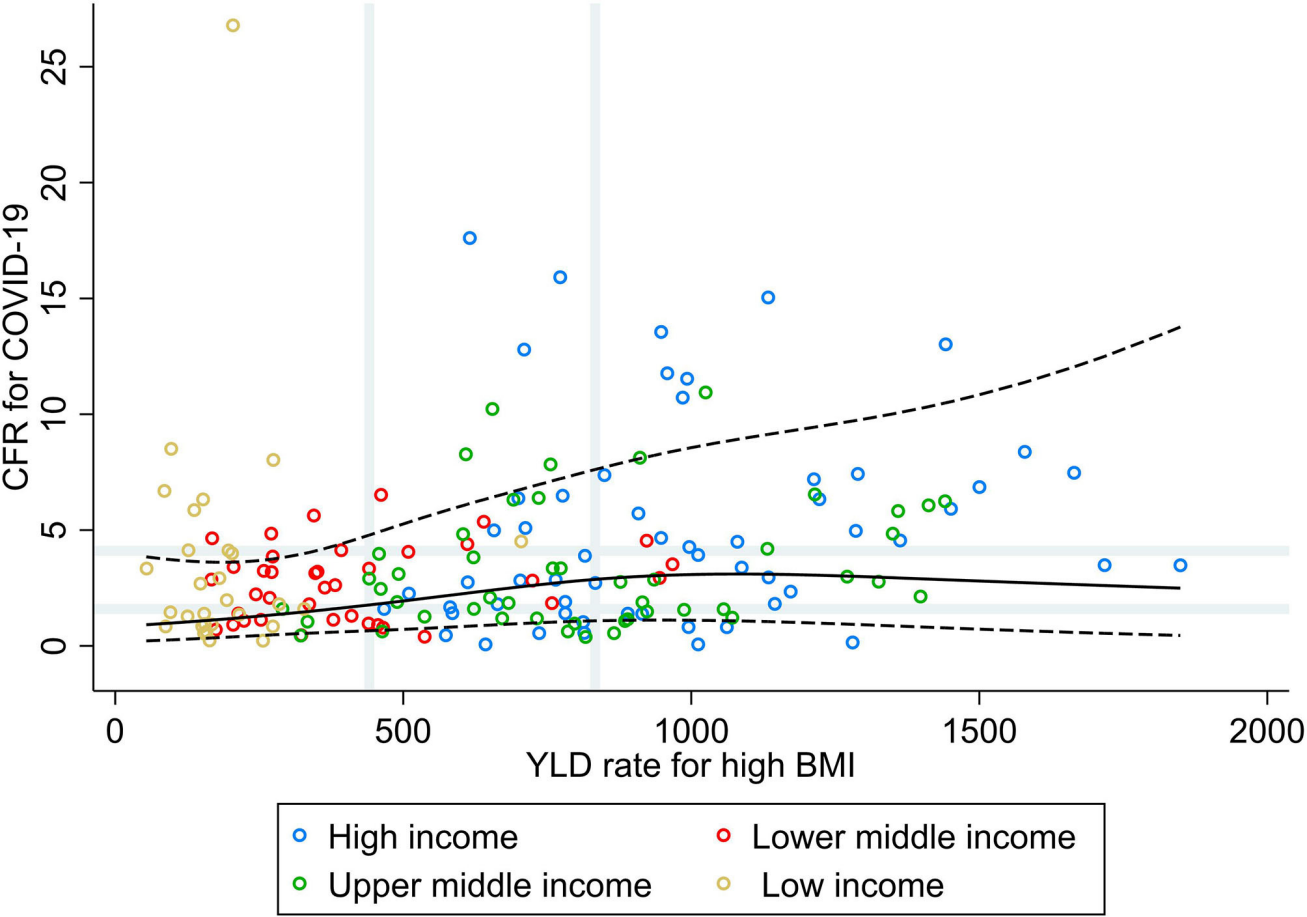
Scatterplot of years lived with disability rate for high BMI against the average case fatality ratio for COVID-19 at week 10 following the first confirmed death reported, stratified by income group (29). All rates described are crude rates per 100,000 population. Gray vertical and horizontal gridlines indicate tertiles dividing lines for the measures: years lived with disability (YLD) rates for high BMI (at 441 and 833) and case fatality ratio for COVID-19 (at 1.6 and 4.1). Solid black line represents the restricted cubic spline, showing the shape of the adjusted association on a continuous scale with knots at the 5th, 35th, 65th, and 95th percentiles (corresponding to YLD rate for high BMI of 148, 461, 814, and 1,412, respectively), with black dashed lines indicating the 95% confidence intervals.

Results from the RCS analysis and the bivariate analyses of YLD rate for high BMI and CFR for COVID-19 (Figure 4) show that countries' vulnerability to fatal COVID-19 was slightly higher with increasing rates of high BMI for countries with low and median rates of high BMI, with no further increases in CFR for COVID-19 for countries with high rates for high BMI. No correlations were seen between the rate of YLD for high BMI and CFR for COVID-19 at the global level, nor at the income group level (ρ < 0.30; Table 1). Increasing rates of high BMI were, however, associated with a higher vulnerability to fatal COVID-19 in LIC (PR 3.31; 95% CI 1.21, 9.05).

### Countries Where Malnutrition Is a Potential Driver for Fatal COVID-19

**Figure 5:**
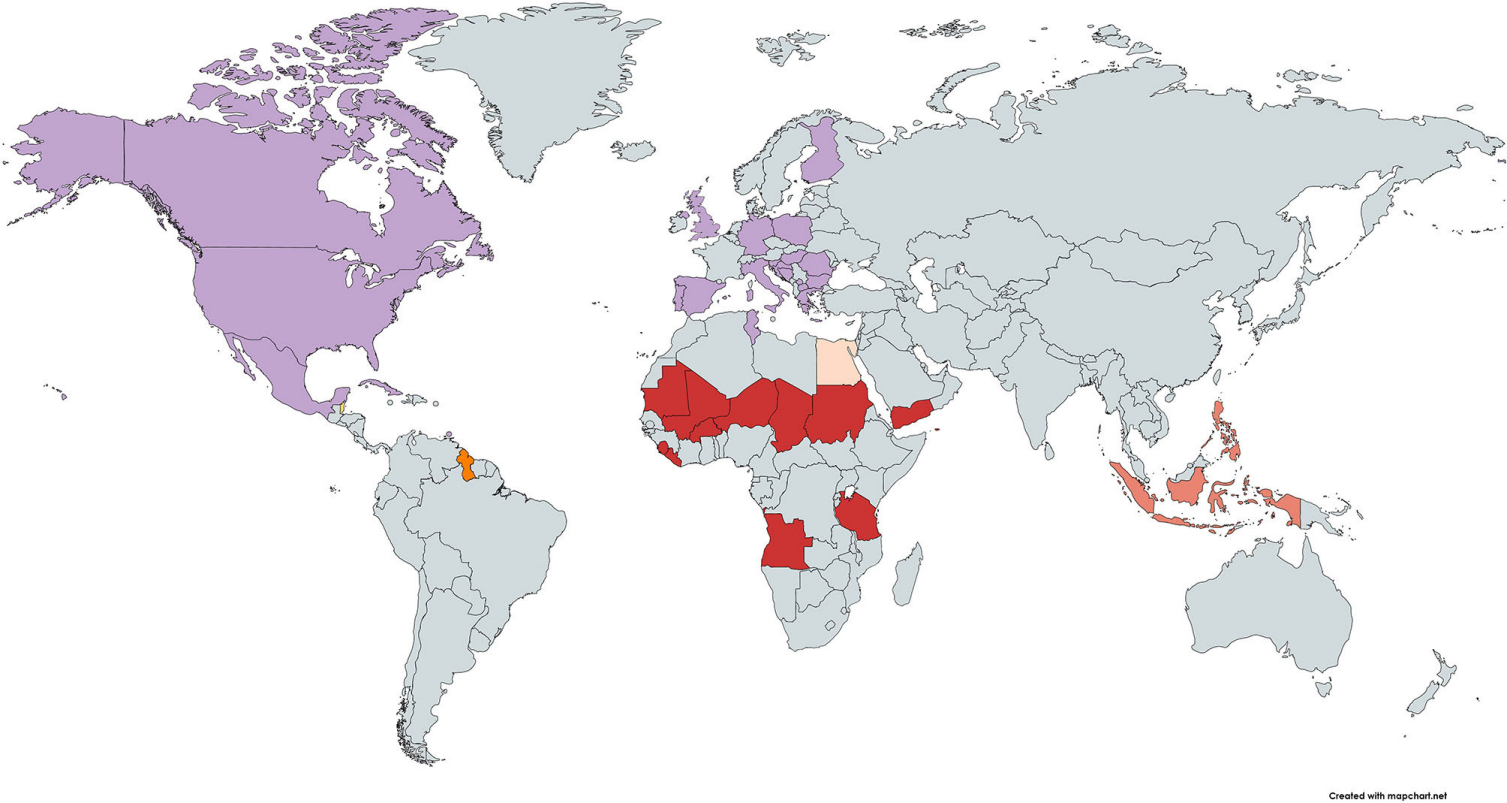
Heatmap of countries where population-level malnutrition appears to be a driver of an increased risk for fatal COVID-19. High (third tertile) CFR at week 10 and 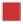 high rates for child growth failure, iron deficiency, and vitamin A deficiency, 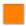 high rates for child growth failure, iron deficiency, and high BMI; 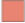 high rates for child growth failure and vitamin A deficiency; 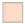 high rates for child growth failure only; 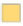 high rates for iron deficiency only; and 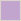 high rates for high BMI only. Note: Countries not on the map with high CFR and high rates for iron deficiencies, vitamin A deficiencies, and BMI were: Fiji; and high rates for high BMI only were: Antigua and Barbuda, Barbados, Bermuda, Northern Mariana Islands, and Virgin Islands (US). CFR, case fatality ratio; BMI, body mass index.

The heatmap for malnutrition shows that malnutrition could be considered as a driver for fatal COVID-19 in 13 countries (7.5%) (Figure 5). No country ranked consistently with high burden on all four malnutrition measures and fatal COVID-19. Countries with high burden of three malnutrition measures and fatal COVID-19 were: Angola, Burkina Faso, Chad, Liberia, Mali, Mauritania, Niger, Sierra Leone, Sudan, Tanzania, and Yemen for high burden of child growth failure and iron and vitamin A deficiencies; Guyana for high burden of child growth failure, iron deficiency, and high BMI; and Fiji for high burden of iron and vitamin A deficiencies and high BMI. When considering only one measure of malnutrition, child growth failure could be considered as a driver for fatal COVID-19 in 15 countries (8.7%), iron deficiencies in 14 countries (8.1%), vitamin A deficiencies in 14 countries (8.1%), and high BMI in 29 countries (16.9%).

### Sensitivity Analyses

Using the CFR at week 15 or the most updated CFR as outcome variable did not alter conclusions of the RCS analyses, the Spearman's rank correlations, and the PRs (results not shown). Countries that were ranked consistently with high burden on at least two malnutrition measures and high burden on fatal COVID-19, as measured by CFR at weeks 10 and 15, and the most updated CFR were: Angola, Burkina Faso, Chad, Guyana, Liberia, Mali, Niger, Sudan, Tanzania, and Yemen. When using CFR at week 15, only Gambia was also additionally included, but Fiji, Mauritania, and Sierra Leone were excluded from the list (results not shown). When using the most updated CFR, the list of countries additionally included Bolivia, Gambia, Malawi, Somalia, Togo, and Zimbabwe.

Furthermore, when considering COVID-19 severity as measured by the daily needs of new ICU beds, countries that have the highest burden of high BMI are experiencing markedly worse COVID-19 severity rates, whereas countries with a high burden of undernutrition did not necessarily have a higher burden of severe COVID-19 cases, as likely related to the lower health care capacity in those countries (Supplementary Figure 2).

## Discussion

Using data on the country-specific burden of malnutrition and fatal COVID-19, the present study identified 10 countries where malnutrition might have played a key role in increasing the country's vulnerability to fatal COVID-19 as suggested by the coexistence of a high burden of malnutrition and elevated mortality among COVID-19 cases. The most affected countries are LIC in sub-Saharan Africa (SSA), such as Angola, Liberia, Tanzania, and particularly among those in the Sahel strip, such as Burkina Faso, Chad, Mali, Niger, and Sudan, as well as Yemen in the Middle East and North African region. Guyana in the Latin America and Caribbean region and Fiji in the East Asia and Pacific region were the only countries vulnerable to fatal COVID-19 through malnutrition, among the list of upper–middle income areas.

These countries that have the highest burden of undernutrition, and particularly high YLD rates for iron deficiency, are also experiencing markedly higher COVID-19 fatality rates. Iron deficiency is generally the consequence of inadequate dietary intake of iron (33), and in particular, an insufficient consumption of animal protein containing high amounts of bioavailable heme iron. A deficiency in iron intake is known to be a strong determinant of anemia, particularly in children and women of reproductive age (34). Although the relationship of anemia with an increased susceptibility to infectious diseases remains controversial and may depend on other immune factors of the community (35), consideration to food and nutrition security are especially needed for those countries lacking access to optimal diets (36). Indeed, albeit not a clear relationship between the burden of vitamin A deficiency and COVID-19 mortality was observed in this study, it is likely that this frequent condition adds to the influence of the burden of undernutrition on COVID-19 fatality, especially in SSA where the prevalence of vitamin A deficiency is the highest (24). Vitamin A deficiency is associated with an increased risk of mortality from measles and diarrhea in children, and although the numbers have decreased globally in the last two decades (37), it has not been the case for the SSA region where this deficiency accounts to 2% of child deaths (24).

Furthermore, considering a broader concept of malnutrition, other dietary risks should be taken into account when looking at the overall impact of suboptimal diets in the vulnerability to COVID-19. While chronic deficiency of essential vitamins and minerals (micronutrients) driven by hunger is a major concern, particularly in SSA (38), global nutrition transition and the spreading prevalence of suboptimal diets high in sodium and low in minimally processed plant-based foods, such as nuts and seeds, rich in omega-3 fatty acids have been consistently identified as major risk factors of the burden of chronic non-communicable diseases (NCDs) (39). This transition towards unhealthier diets is evidenced, for example, in the markedly different food intakes observed in southern SSA compared with other SSA regions in terms of lower consumption of fruits and legumes, increased intake of red meat and sugary drinks, and elevated ratio between omega-6/omega-3 fatty acids (39), similar to the levels observed in Western diets. An increased omega-6/omega-3 ratio has been associated with an elevated risk of cardiovascular, inflammatory, and autoimmune diseases (40).

Factors, such as international exposure, size of urban population, and strength of the health system, were initially identified as determinants of COVID-19 cases in Africa (41). In SSA countries, however, preventive measures, such as self-isolation at home, particularly made COVID-19 to exacerbate food insecurity as a result of lower purchase power due to unemployment and pressure on the planting season due to a lack of inputs and labor (36) resulting in lower food production, as reported in Nigeria (42) and Bangladesh (43), and likely mirroring the situation across most parts of the African continent (44). In particular, the Sahel strip, one of the world's areas most affected by hunger before COVID-19, is now facing exceptional high levels of food insecurity with the number of food insecure people doubling or even tripling during the pandemic (45, 46). Early estimates also suggested that low- and middle-income countries should anticipate indirect large increases in childhood undernutrition and in maternal and child deaths resulting from the reduced access to food and the widespread disruption of the health systems due to COVID-19 (47, 48). Although the favorable clinical course of COVID-19 in children (49), the indirect effects of COVID-19 are likely to have a long-lasting impact on children in LIC, because of increasing poverty levels, disrupted schooling and lack of access to school feeding schemes, reduced access to health facilities, and interruptions in vaccination and other child health programs (50). This underlines the fact that malnutrition is the outcome of a disturbed interplay between the individual and household decision-making, the agri-food, environmental, and health systems at the community and/or national level (51), and has complicated relationships with infectious diseases (15), including COVID-19 (45–48). In future investigations during the coming months and years, it would be important to measure excess mortality for the countries' burden to fatal COVID-19 directly and indirectly, to reveal the real impact of COVID-19 on population health and inform public health measures, such as efforts to minimize disruption to health care and maximize food security, in particular to improve population maternal and child health (MCH).

Findings from the present study show that LIC and lower–middle-income countries are mostly dispersed above the highest tertile line of undernutrition and below the lowest tertile line of overnutrition, and that countries with higher burden of iron deficiencies presented higher CFR, most clearly for LIC. Contrastingly, our results indicate that HIC are mostly burdened by elevated rates of high BMI, whereas undernutrition, measured as either child growth failure or iron and vitamin A deficiencies, is much less prevalent in comparison with low- and middle-income countries. None of the measures, however, seem to be clearly related with greater vulnerability to COVID-19, neither at the global level nor at the income group level. At the individual level, however, evidence shows that obesity (high BMI) has been associated with severe COVID-19 (4, 5). In addition, health disparities in diet and obesity are believed to disproportionately affect the burden of infections, hospitalizations, and deaths from COVID-19 after infection, as seen, for example, in the U.S. where Native and African Americans have a four-to-five times higher hospitalization rates than White Americans (52). As mentioned, other factors, such as aging and health loss due to chronic conditions, also have a substantial impact on COVID-19 severity (26). A previous published modeling study estimated that one in five individuals worldwide has an underlying chronic cause of disease that could predispose them to severe COVID-19 if infected, with a higher share of the population at increased risk for Europe, Central Asia, and North America (53). Yet, a number of other important factors related to the management of the pandemic will also contribute to the extent of fatal COVID-19, including, but not limited to: (sub)national responses to the outbreak through public health non-pharmaceutical practices of handwashing, physical distancing, and the use of facemasks; health care access and capacities; and population density. Thus, a complete risk assessment of a countries' vulnerability to COVID-19 should take into account all elements that play a role in this pandemic.

Undernutrition early in life is a risk factor for overnutrition in adulthood, hence rising concerns for countries with a high prevalence of early life undernutrition being at higher risk for being affected by multiple burdens of malnutrition (54, 55). The present paper addresses double and triple burdens of malnutrition by including measures of malnutrition both for MCH and for NCDs. Malnutrition for MCH has been traditionally considered as insufficient caloric or nutrient intake, leading to child growth failure (i.e., insufficient physical growth, rapid weight loss, or failure to gain weight) and exacerbations of iron deficiency anemia, whereas malnutrition for NCDs as an excess consumption of calories, leading to high BMI. Countries' burden of malnutrition, as well as the burden of other conditions related to worse COVID-19 prognosis, was quantified using estimates from the GBD 2019 (20, 21), therefore using the most updated data, almost concurrent to the ongoing pandemic. Because of our aim to identify countries at high risk for fatal COVID-19 due to malnutrition, we opted for crude rates to more fairly represent countries' demographics, also considering that age and sex distributions influence the risk for fatal COVID-19, which is higher among older populations (53). Previous analysis have also acknowledged, by using crude rates of either prevalence (53) or YLD (56) for COVID-19 vulnerable health conditions, the importance of identifying high-risk countries and high-risk groups within countries to mitigate the COVID-19 impact and inform public health strategies to shield those at the highest risk.

Countries' burden of COVID-19 fatality was quantified using CFR for COVID-19 at week 10 following the first confirmed death reported as a proxy for severity of the disease outcome, instead of infection fatality ratio (IFR), the more preferred measure to assess the severity of epidemics (57). As IFR estimates the proportion of deaths among all infected individuals, seroprevalence studies representative for the general population are needed to capture the undiagnosed asymptomatic and mildly symptomatic infections. The few available seroprevalence studies, mainly from HIC and China, point to a median global IFR of 0.27% with large variability across locations depending on population age-structure, and the case-mix of infected and deceased patients, as well as other local factors, such as population burden of underlying comorbidities, public health measures, and health care (58–60). There are indeed an elevated number of undetected cases for COVID-19 making CFR overestimating the true disease fatality. In any case, measures for disease fatality should be interpreted with caution because of its dependence on a particular context, time, and place related to the health care and testing capacity, the reporting practices of COVID-19 cases and deaths, the number of confirmed cases (for CFR), the number of infected individuals detected (for IFR), the number of confirmed deaths, the standard care response for suspected and confirmed COVID-19 patients, the COVID-19 patients' ability to recover, etc. In particular, with countries facing different stages of the pandemic at a particular time (61) and having different levels of testing efforts (30, 62), comparability of the fatality measures between countries might be limited. The present analysis used for each country the CFR measured at week 10 following the first death reported of a confirmed case in that country, as a way to avert the effect of the different stages of the pandemic on the calculation of CFR (31). Furthermore, sensitivity analyses also suggest robust findings, as using for each country CFR at week 15 and the most updated shows similar results. In secondary analyses, we used the cumulative number of ICU beds use from the first COVID-19 death reported up until 10 weeks later, as a proxy for COVID-19 severity at the country level. This measure is, however, not available for all countries (81%) and provides only a partial picture of the relationship between malnutrition and COVID-19's burdens. Countries with the greatest burden of high BMI are reporting markedly worse COVID-19 severity rates, thus confirming the relationship observed for CFR. However, countries where undernutrition is highly prevalent did not appear to be related with the higher use of ICU beds, likely reflecting a limited health system's capacity in those countries. The use of CFR is commonly regarded as a poor measure of mortality risk of COVID-19 throughout the early and middle stages of an ongoing outbreak, as it might under- or overestimate the true ratio between confirmed deaths and confirmed cases. However, due to the inconsistent reporting of other indicators, CFR represents the most comprehensive measures at the present time of the pandemic. With the accumulation of information about cases at the end of the pandemic, the estimate will be close to those finally observed once the epidemic recedes (63). Repeating the analyses at the end of the pandemic might further enlighten the role played by malnutrition on fatal COVID-19, and depending on the countries' reporting practices and availability of seroprevalence studies in the general population, the IFR could be used as a more accurate measure of disease fatality.

In conclusion, population-level malnutrition appears to be a driver of an increased risk for fatal COVID-19 in areas with marked burden of undernutrition as those in the Sahel strip. Using routine estimates of the risk factor burden allowed the rapid assessment for countries' vulnerability to COVID-19 due to malnutrition and identifying those countries where malnutrition is a virulence factor for severe COVID-19. Our findings are relevant to country-level, global interpretation, and cannot be extrapolated to high-risk individuals or populations within particular countries because of ecological fallacy. To mitigate the impact of severe COVID-19, preventive public health strategies of the response plans to COVID-19 should also pay attention to food, nutrition, and integrate sustainable food systems interventions to promote the production, distribution, and consumption of healthy diets, along with social protection, with the aim to improve the nutritional status of the population, especially those at higher risk, during and at the end of the pandemic (64).

## Data Availability Statement

Publicly available datasets were analyzed in this study. This data can be found here: http://ghdx.healthdata.org/gbd-results-tool; https://ourworldindata.org/coronavirus; https://datahelpdesk.worldbank.org/knowledgebase/articles/906519-world-bank-country-and-lending-groups; https://population.un.org/wpp/Download/Standard/Population/.

## Author Contributions

JP and EM conceptualized and designed the study, identified the relevant data sources, retrieved the data, wrote the manuscript, and revised, read, and approved the submitted version. EM performed the statistical analyses. All authors contributed to the article and approved the submitted version.

## Conflict of Interest

The authors declare that the research was conducted in the absence of any commercial or financial relationships that could be construed as a potential conflict of interest.
