# Supplementary material for: The Burden of Malnutrition and Fatal COVID-19: A Global Burden of Disease Analysis
Source: Front Nutr. 2021 Jan 21;7:619850. doi: 10.3389/fnut.2020.619850 (PMC7858665; doi:10.3389/fnut.2020.619850)
Supplement: Supplementary file 1 [file Data_Sheet_1.docx]

**Supplementary Table 1** Countries’ burden to malnutrition and to COVID-19, for each country ordered by income group and CFR at week 10 following first confirmed death reported.

| **Country** | **iso** | | **Region** | | **Malnutrition** | | | | **COVID-19 at week 10** | | |
| --- | --- | --- | --- | --- | --- | --- | --- | --- | --- | --- | --- |
|  |  |  |  |  | **Death rate** | **YLD rate** | | | **Per million** | | **CFR** |
|  |  |  |  |  | **Child growth failure** | **Deficiencies** | | **High BMI** | **Total cases** | **Total deaths** |  |
|  |  |  |  |  |  | **Iron** | **Vit A** |  |  |  |  |
| **High income countries** | | | | | | | | | | | |
| France | FRA | | ECA | | 6.9 | 22 | 0.0 | 615 | 1763 | 310.7 | 17.6 |
| Belgium | BEL | | ECA | | 3.0 | 28 | 0.1 | 772 | 4803 | 764.7 | 15.9 |
| United Kingdom | GBR | | ECA | | 0.2 | 66 | 0.2 | 1133 | 3118 | 469.0 | 15.0 |
| Italy | ITA | | ECA | | 1.0 | 32 | 0.2 | 948 | 3333 | 451.9 | 13.6 |
| Hungary | HUN | | ECA | | 0.3 | 101 | 1.4 | 1441 | 377 | 49.0 | 13.0 |
| Netherlands | NLD | | ECA | | 0.8 | 28 | 0.1 | 710 | 2498 | 319.6 | 12.8 |
| Spain | ESP | | ECA | | 0.7 | 47 | 0.1 | 958 | 4832 | 568.9 | 11.8 |
| Antigua and Barbuda | ATG | | LAC | | 3.6 | 260 | 4.4 | 993 | 266 | 30.6 | 11.5 |
| Bahamas. The | BHS | | LAC | | 1.9 | 299 | 4.3 | 985 | 261 | 28.0 | 10.7 |
| Virgin Islands (U.S.) | VIR | | LAC | | 3.7 | 239 | 0.5 | 1579 | 685 | 57.5 | 8.4 |
| Northern Mariana Islands | MNP | | EAP | | 3.2 | 236 | 2.6 | 1664 | 467 | 34.7 | 7.5 |
| Barbados | BRB | | LAC | | 2.1 | 176 | 2.7 | 1289 | 328 | 24.4 | 7.4 |
| Canada | CAN | | NA | | 0.9 | 38 | 0.1 | 849 | 1947 | 143.7 | 7.4 |
| Slovenia | SVN | | ECA | | 0.1 | 85 | 0.9 | 1213 | 706 | 50.9 | 7.2 |
| Trinidad and Tobago | TTO | | LAC | | 2.4 | 302 | 4.2 | 1500 | 83 | 5.7 | 6.9 |
| Andorra | AND | | ECA | | 0.2 | 30 | 0.1 | 777 | 10197 | 660.1 | 6.5 |
| Ireland | IRL | | ECA | | 0.2 | 29 | 0.1 | 700 | 4855 | 309.7 | 6.4 |
| Bermuda | BMU | | NA | | 0.7 | 121 | 0.3 | 1222 | 2280 | 144.5 | 6.3 |
| United States | USA | | NA | | 1.8 | 66 | 0.1 | 1451 | 3645 | 215.9 | 5.9 |
| Greece | GRC | | ECA | | 0.1 | 43 | 0.2 | 908 | 270 | 15.4 | 5.7 |
| Switzerland | CHE | | ECA | | 0.9 | 27 | 0.1 | 712 | 3490 | 177.9 | 5.1 |
| Denmark | DNK | | ECA | | 0.9 | 32 | 0.1 | 657 | 1918 | 95.8 | 5.0 |
| Poland | POL | | ECA | | 0.4 | 136 | 1.5 | 1286 | 492 | 24.4 | 5.0 |
| Finland | FIN | | ECA | | 0.1 | 33 | 0.1 | 947 | 1201 | 56.0 | 4.7 |
| Croatia | HRV | | ECA | | 0.1 | 88 | 1.1 | 1362 | 547 | 24.9 | 4.6 |
| Germany | DEU | | ECA | | 0.7 | 31 | 0.1 | 1080 | 2063 | 92.8 | 4.5 |
| Portugal | PRT | | ECA | | 1.2 | 39 | 0.2 | 996 | 2957 | 126.4 | 4.3 |
| Lithuania | LTU | | ECA | | 0.2 | 152 | 0.1 | 1012 | 599 | 23.6 | 3.9 |
| Austria | AUT | | ECA | | 0.1 | 31 | 0.1 | 815 | 1794 | 69.9 | 3.9 |
| Czech Republic | CZE | | ECA | | 1.2 | 96 | 1.2 | 1717 | 849 | 29.7 | 3.5 |
| Puerto Rico | PRI | | LAC | | 3.1 | 139 | 0.3 | 1849 | 1372 | 47.7 | 3.5 |
| Estonia | EST | | ECA | | 0.2 | 112 | 0.1 | 1087 | 1403 | 47.5 | 3.4 |
| Guam | GUM | | EAP | | 3.9 | 297 | 3.2 | 1134 | 1000 | 29.6 | 3.0 |
| Panama | PAN | | LAC | | 5.6 | 202 | 5.1 | 764 | 2149 | 61.5 | 2.9 |
| Norway | NOR | | ECA | | 1.8 | 31 | 0.1 | 703 | 1516 | 42.9 | 2.8 |
| Uruguay | URY | | LAC | | 3.5 | 121 | 3.0 | 612 | 239 | 6.6 | 2.8 |
| Luxembourg | LUX | | ECA | | 1.2 | 30 | 0.1 | 833 | 6324 | 172.1 | 2.7 |
| Latvia | LVA | | ECA | | 0.2 | 145 | 0.1 | 1172 | 577 | 13.6 | 2.3 |
| Korea. Rep. | KOR | | EAP | | 0.3 | 136 | 0.1 | 510 | 209 | 4.7 | 2.3 |
| New Zealand | NZL | | EAP | | 0.4 | 66 | 0.2 | 781 | 239 | 4.6 | 1.9 |
| Slovak Republic | SVK | | ECA | | 0.4 | 115 | 1.4 | 1145 | 282 | 5.1 | 1.8 |
| Cyprus | CYP | | ECA | | 0.5 | 28 | 0.2 | 664 | 1076 | 19.4 | 1.8 |
| Israel | ISR | | MENA | | 0.6 | 53 | 2.5 | 581 | 1935 | 32.4 | 1.7 |
| Japan | JPN | | EAP | | 1.2 | 128 | 0.2 | 467 | 81 | 1.3 | 1.6 |
| Brunei Darussalam | BRN | | EAP | | 0.6 | 192 | 0.6 | 585 | 322 | 4.6 | 1.4 |
| Taiwan. China | TWN | | EAP | | 0.6 | 93 | 0.2 | 781 | 18 | 0.3 | 1.4 |
| Australia | AUS | | EAP | | 0.6 | 56 | 0.0 | 890 | 269 | 3.8 | 1.4 |
| Malta | MLT | | MENA | | 0.3 | 41 | 0.2 | 915 | 1469 | 20.4 | 1.4 |
| Chile | CHL | | LAC | | 3.2 | 43 | 0.4 | 812 | 4303 | 44.5 | 1.0 |
| Kuwait | KWT | | MENA | | 0.4 | 175 | 4.5 | 995 | 7755 | 63.5 | 0.8 |
| United Arab Emirates | ARE | | MENA | | 0.1 | 144 | 0.3 | 1062 | 3138 | 25.5 | 0.8 |
| Saudi Arabia | SAU | | MENA | | 0.4 | 62 | 0.1 | 814 | 2351 | 13.3 | 0.6 |
| Iceland | ISL | | ECA | | 0.5 | 27 | 0.1 | 736 | 5286 | 29.3 | 0.6 |
| Oman | OMN | | MENA | | 0.7 | 183 | 11.7 | 574 | 2969 | 13.8 | 0.5 |
| Bahrain | BHR | | MENA | | 0.4 | 135 | 0.7 | 1280 | 4797 | 7.3 | 0.2 |
| Singapore | SGP | | EAP | | 0.1 | 107 | 0.1 | 643 | 5542 | 3.9 | 0.1 |
| Qatar | QAT | | MENA | | 0.2 | 71 | 0.2 | 1012 | 20938 | 14.7 | 0.1 |
| **Upper-middle income countries** | | | | | | | | | | | |
| Mexico | MEX | LAC | | | 7.0 | 114 | 9.2 | 1025 | 556 | 60.9 | 10.9 |
| Belize | BLZ | LAC | | | 4.8 | 378 | 8.7 | 655 | 49 | 5.0 | 10.2 |
| Ecuador | ECU | LAC | | | 5.8 | 96 | 5.6 | 609 | 1905 | 157.8 | 8.3 |
| Guyana | GUY | LAC | | | 9.1 | 501 | 9.2 | 911 | 157 | 12.7 | 8.1 |
| Algeria | DZA | MENA | | | 1.9 | 188 | 7.6 | 756 | 160 | 12.5 | 7.8 |
| Romania | ROU | ECA | | | 1.4 | 147 | 2.2 | 1214 | 967 | 63.3 | 6.5 |
| Brazil | BRA | LAC | | | 5.5 | 306 | 8.7 | 735 | 1539 | 98.2 | 6.4 |
| Iran. Islamic Rep. | IRN | MENA | | | 0.8 | 98 | 0.7 | 691 | 1063 | 67.2 | 6.3 |
| Fiji | FJI | EAP | | | 7.4 | 499 | 13.5 | 1440 | 36 | 2.2 | 6.3 |
| Bosnia and Herzegovina | BIH | ECA | | | 0.1 | 162 | 2.4 | 1412 | 740 | 44.9 | 6.1 |
| North Macedonia | MKD | ECA | | | 0.3 | 140 | 3.8 | 1359 | 988 | 57.6 | 5.8 |
| Bulgaria | BGR | ECA | | | 0.6 | 152 | 2.3 | 1350 | 315 | 15.3 | 4.9 |
| Argentina | ARG | LAC | | | 3.5 | 155 | 4.6 | 604 | 157 | 7.6 | 4.8 |
| Cuba | CUB | LAC | | | 0.7 | 194 | 0.8 | 1132 | 170 | 7.2 | 4.2 |
| China | CHN | EAP | | | 1.5 | 85 | 2.9 | 458 | 56 | 2.2 | 4.0 |
| Iraq | IRQ | MENA | | | 2.3 | 174 | 8.3 | 622 | 73 | 2.8 | 3.8 |
| Albania | ALB | ECA | | | 1.5 | 180 | 5.1 | 773 | 321 | 10.8 | 3.4 |
| Colombia | COL | LAC | | | 3.9 | 88 | 4.0 | 759 | 456 | 15.3 | 3.4 |
| Dominican Republic | DOM | LAC | | | 6.3 | 235 | 7.1 | 492 | 1382 | 42.9 | 3.1 |
| Mauritius | MUS | SSA | | | 1.9 | 204 | 3.0 | 1270 | 263 | 7.9 | 3.0 |
| Peru | PER | LAC | | | 5.0 | 212 | 9.6 | 441 | 3665 | 106.7 | 2.9 |
| Lebanon | LBN | MENA | | | 1.0 | 81 | 1.2 | 935 | 133 | 3.8 | 2.9 |
| Montenegro | MNE | ECA | | | 0.2 | 130 | 1.7 | 1325 | 516 | 14.3 | 2.8 |
| Turkey | TUR | ECA | | | 1.4 | 153 | 1.4 | 877 | 1846 | 51.1 | 2.8 |
| Botswana | BWA | SSA | | | 22.0 | 318 | 21.3 | 461 | 17 | 0.4 | 2.5 |
| Serbia | SRB | ECA | | | 0.2 | 137 | 4.6 | 1398 | 1643 | 35.1 | 2.1 |
| South Africa | ZAF | SSA | | | 13.6 | 248 | 9.7 | 651 | 549 | 11.5 | 2.1 |
| Guatemala | GTM | LAC | | | 21.5 | 245 | 10.7 | 489 | 133 | 2.5 | 1.9 |
| Libya | LBY | MENA | | | 0.8 | 181 | 6.3 | 915 | 40 | 0.7 | 1.9 |
| Thailand | THA | EAP | | | 1.8 | 114 | 3.6 | 683 | 43 | 0.8 | 1.9 |
| Malaysia | MYS | EAP | | | 1.3 | 285 | 0.4 | 622 | 221 | 3.5 | 1.6 |
| Equatorial Guinea | GNQ | SSA | | | 7.0 | 386 | 24.0 | 290 | 1426 | 22.8 | 1.6 |
| Georgia | GEO | ECA | | | 0.8 | 332 | 1.3 | 1056 | 207 | 3.3 | 1.6 |
| Jamaica | JAM | LAC | | | 2.4 | 291 | 4.9 | 987 | 195 | 3.0 | 1.6 |
| Armenia | ARM | ECA | | | 1.7 | 236 | 0.2 | 923 | 3285 | 49.2 | 1.5 |
| Paraguay | PRY | LAC | | | 5.8 | 253 | 9.3 | 537 | 122 | 1.5 | 1.3 |
| Suriname | SUR | LAC | | | 4.8 | 363 | 8.7 | 1071 | 216 | 2.7 | 1.2 |
| Jordan | JOR | MENA | | | 1.8 | 195 | 7.9 | 672 | 74 | 0.9 | 1.2 |
| Azerbaijan | AZE | ECA | | | 7.0 | 325 | 1.5 | 732 | 422 | 5.0 | 1.2 |
| Russian Federation | RUS | ECA | | | 0.6 | 149 | 0.0 | 889 | 2780 | 32.2 | 1.2 |
| Costa Rica | CRI | LAC | | | 0.8 | 107 | 3.7 | 885 | 181 | 2.0 | 1.1 |
| Namibia | NAM | SSA | | | 20.9 | 348 | 17.8 | 334 | 3852 | 40.6 | 1.1 |
| Venezuela. RB | VEN | LAC | | | 3.9 | 107 | 5.2 | 797 | 56 | 0.5 | 1.0 |
| Sri Lanka | LKA | SA | | | 1.2 | 226 | 4.2 | 786 | 80 | 0.5 | 0.6 |
| Gabon | GAB | SSA | | | 9.1 | 629 | 17.7 | 463 | 945 | 5.9 | 0.6 |
| Belarus | BLR | ECA | | | 0.4 | 130 | 0.2 | 866 | 5140 | 28.6 | 0.6 |
| Maldives | MDV | SA | | | 1.7 | 214 | 6.0 | 322 | 4552 | 20.6 | 0.5 |
| Kazakhstan | KAZ | ECA | | | 2.1 | 384 | 7.0 | 816 | 641 | 2.5 | 0.4 |
| **Lower-middle income countries** | | | | | | | | | | | |
| Indonesia | IDN | | | EAP | 12.5 | 289 | 16.8 | 461 | 64 | 4.2 | 6.5 |
| Philippines | PHL | | | EAP | 11.8 | 193 | 15.6 | 345 | 41 | 2.3 | 5.6 |
| Egypt. Arab Rep. | EGY | | | MENA | 9.5 | 223 | 4.4 | 640 | 103 | 5.5 | 5.4 |
| Mauritania | MRT | | | SSA | 28.3 | 670 | 36.5 | 271 | 210 | 10.2 | 4.9 |
| Angola | AGO | | | SSA | 39.2 | 435 | 45.5 | 168 | 3 | 0.1 | 4.7 |
| Tunisia | TUN | | | MENA | 0.7 | 96 | 4.3 | 922 | 89 | 4.0 | 4.5 |
| Nicaragua | NIC | | | LAC | 5.3 | 87 | 4.0 | 611 | 138 | 6.0 | 4.4 |
| Sudan | SDN | | | SSA | 18.5 | 385 | 26.2 | 392 | 61 | 2.5 | 4.1 |
| Honduras | HND | | | LAC | 5.6 | 221 | 8.3 | 509 | 527 | 21.4 | 4.1 |
| Zimbabwe | ZWE | | | SSA | 41.3 | 415 | 44.3 | 273 | 9 | 0.3 | 3.9 |
| Moldova | MDA | | | ECA | 0.5 | 239 | 0.4 | 967 | 1723 | 60.8 | 3.5 |
| Kenya | KEN | | | SSA | 26.0 | 313 | 47.2 | 205 | 36 | 1.2 | 3.4 |
| Bolivia | BOL | | | LAC | 14.4 | 436 | 15.1 | 440 | 997 | 33.4 | 3.3 |
| Cameroon | CMR | | | SSA | 57.2 | 453 | 58.7 | 258 | 214 | 6.9 | 3.2 |
| Congo. Rep. | COG | | | SSA | 20.7 | 530 | 73.6 | 351 | 118 | 3.8 | 3.2 |
| Vietnam | VNM | | | EAP | 1.7 | 143 | 3.1 | 271 | 11 | 0.4 | 3.2 |
| India | IND | | | SA | 9.0 | 844 | 21.7 | 347 | 70 | 2.2 | 3.1 |
| Ukraine | UKR | | | ECA | 0.4 | 100 | 0.2 | 945 | 469 | 13.8 | 2.9 |
| Nigeria | NGA | | | SSA | 96.4 | 864 | 25.2 | 167 | 44 | 1.3 | 2.9 |
| Morocco | MAR | | | MENA | 3.8 | 247 | 9.8 | 724 | 183 | 5.2 | 2.8 |
| Lesotho | LSO | | | SSA | 38.4 | 389 | 34.0 | 382 | 581 | 15.3 | 2.6 |
| Myanmar | MMR | | | EAP | 12.0 | 539 | 14.1 | 364 | 4 | 0.1 | 2.5 |
| Comoros | COM | | | SSA | 30.1 | 628 | 31.4 | 244 | 361 | 8.1 | 2.2 |
| Pakistan | PAK | | | SA | 28.8 | 824 | 19.4 | 268 | 254 | 5.3 | 2.1 |
| El Salvador | SLV | | | LAC | 6.2 | 147 | 7.9 | 758 | 441 | 8.2 | 1.9 |
| São Tomé and Principe | STP | | | SSA | 8.7 | 455 | 37.4 | 337 | 3288 | 59.3 | 1.8 |
| Bangladesh | BGD | | | SA | 6.3 | 463 | 9.1 | 214 | 225 | 3.2 | 1.4 |
| Papua New Guinea | PNG | | | EAP | 49.1 | 543 | 24.2 | 410 | 60 | 0.8 | 1.3 |
| Kyrgyz Republic | KGZ | | | ECA | 4.2 | 413 | 10.3 | 378 | 306 | 3.5 | 1.1 |
| Senegal | SEN | | | SSA | 27.7 | 892 | 42.2 | 253 | 253 | 2.9 | 1.1 |
| Côte d'Ivoire | CIV | | | SSA | 32.9 | 679 | 56.9 | 223 | 120 | 1.3 | 1.1 |
| Cabo Verde | CPV | | | SSA | 4.3 | 427 | 12.0 | 440 | 742 | 7.2 | 1.0 |
| Djibouti | DJI | | | MENA | 39.6 | 547 | 29.5 | 205 | 4531 | 41.6 | 0.9 |
| Eswatini | SWZ | | | SSA | 28.5 | 294 | 21.2 | 456 | 566 | 5.2 | 0.9 |
| West Bank and Gaza | PSE | | | MENA | 1.0 | 175 | 12.2 | 465 | 124 | 1.0 | 0.8 |
| Zambia | ZMB | | | SSA | 37.7 | 1105 | 44.9 | 174 | 64 | 0.5 | 0.7 |
| Ghana | GHA | | | SSA | 20.2 | 544 | 45.7 | 323 | 227 | 1.1 | 0.5 |
| Uzbekistan | UZB | | | ECA | 10.8 | 595 | 9.5 | 537 | 113 | 0.5 | 0.4 |
| **Low income countries** | | | | | | | | | | | |
| Yemen. Rep. | YEM | | | MENA | 25.2 | 939 | 52.3 | 204 | 43 | 11.4 | 26.8 |
| Chad | TCD | | | SSA | 214.0 | 866 | 126.6 | 97 | 53 | 4.5 | 8.5 |
| Liberia | LBR | | | SSA | 37.0 | 372 | 37.9 | 274 | 76 | 6.1 | 8.0 |
| Niger | NER | | | SSA | 182.8 | 821 | 171.4 | 85 | 39 | 2.6 | 6.7 |
| Burkina Faso | BFA | | | SSA | 111.0 | 1024 | 110.1 | 152 | 39 | 2.5 | 6.3 |
| Mali | MLI | | | SSA | 136.3 | 1256 | 124.8 | 137 | 72 | 4.2 | 5.9 |
| Syrian Arab Republic | SYR | | | MENA | 2.1 | 199 | 3.4 | 705 | 7 | 0.3 | 4.5 |
| Sierra Leone | SLE | | | SSA | 63.4 | 712 | 83.9 | 127 | 179 | 7.4 | 4.1 |
| Tanzania | TZA | | | SSA | 37.0 | 724 | 40.1 | 196 | 9 | 0.4 | 4.1 |
| Gambia. The | GMB | | | SSA | 20.1 | 992 | 77.7 | 203 | 10 | 0.4 | 4.0 |
| Somalia | SOM | | | SSA | 148.1 | 816 | 140.8 | 54 | 164 | 5.5 | 3.3 |
| Togo | TGO | | | SSA | 52.1 | 667 | 64.3 | 181 | 54 | 1.6 | 2.9 |
| Congo. Dem. Rep. | COD | | | SSA | 35.2 | 568 | 86.6 | 148 | 28 | 0.7 | 2.7 |
| South Sudan | SSD | | | SSA | 96.6 | 694 | 54.0 | 194 | 196 | 3.9 | 2.0 |
| Afghanistan | AFG | | | SA | 36.3 | 285 | 51.2 | 284 | 337 | 6.1 | 1.8 |
| Haiti | HTI | | | LAC | 38.6 | 702 | 33.0 | 328 | 343 | 5.5 | 1.6 |
| Ethiopia | ETH | | | SSA | 38.9 | 491 | 61.0 | 96 | 22 | 0.3 | 1.5 |
| Guinea-Bissau | GNB | | | SSA | 56.3 | 778 | 83.9 | 154 | 847 | 11.8 | 1.4 |
| Benin | BEN | | | SSA | 65.3 | 526 | 90.0 | 215 | 29 | 0.4 | 1.4 |
| Central African Republic | CAF | | | SSA | 146.8 | 599 | 111.7 | 125 | 953 | 12.2 | 1.3 |
| Uganda | UGA | | | SSA | 30.9 | 432 | 31.3 | 155 | 170 | 1.6 | 0.9 |
| Malawi | MWI | | | SSA | 33.8 | 700 | 61.3 | 167 | 26 | 0.2 | 0.9 |
| Madagascar | MDG | | | SSA | 70.1 | 516 | 45.6 | 151 | 288 | 2.5 | 0.9 |
| Burundi | BDI | | | SSA | 68.8 | 473 | 51.9 | 87 | 10 | 0.1 | 0.8 |
| Tajikistan | TJK | | | ECA | 19.7 | 358 | 14.8 | 273 | 667 | 5.6 | 0.8 |
| Mozambique | MOZ | | | SSA | 38.6 | 690 | 80.1 | 159 | 58 | 0.4 | 0.6 |
| Guinea | GIN | | | SSA | 73.9 | 667 | 83.1 | 154 | 375 | 2.1 | 0.5 |
| Rwanda | RWA | | | SSA | 27.3 | 337 | 22.1 | 164 | 161 | 0.4 | 0.2 |
| Nepal | NPL | | | SA | 10.8 | 568 | 11.2 | 256 | 616 | 1.4 | 0.2 |

*According to World Bank 2019 [27]. Abbreviations: BMI, body mass index; CFR, case fatality ratio; Death rate, rate of death per 100.000 for all ages; EAP, East Asia & Pacific; ECA, Europe & Central Asia; LAC, Latin America & Caribbean; MENA, Middle East & North Africa; NA, North America; SA, South Asia; SSA, Sub-Saharan Africa; YLD rate, rate of Years Lived with Disability per 100.000 for all ages.

1A: YLD rates for COVID-19 related vulnerable health conditions, as identified by WHO [25]

1B: Percentage of population aged 65 years and older

**Supplementary Figures 1** Scatterplots of recognized sources of vulnerability to severe or fatal COVID-19 against the average case fatality ratio for COVID-19 at week 10 following first confirmed death reported.

*All rates described are crude rates per 100,000 population. Grey vertical and horizontal gridlines indicate tertiles dividing lines for the measures: Years Lived with Disability (YLD) rates for COVID-19 related vulnerable health conditions (at 1398 and 2184) for 1A, % of aged 65 and older (at 4 and 11) for 1B, and case fatality ratio for COVID-19 (at 1.6 and 4.1).*

**Supplementary Figure 2A** Death rate for child growth failure

*All rates described are crude rates per 100,000 population. Grey vertical and horizontal gridlines indicate tertiles dividing lines for the measures: death rates for child growth failure (at 1.0 and 6.3) and cumulative of new ICU bed for COVID-19 (at 3.4 and 11.2). Solid black line represents the restricted cubic spline, showing the shape of the adjusted association on a continuous scale with knots at the 5th, 35th, 65th and 95th percentiles (corresponding to death rate for child growth failure of 0.1, 1.0, 6.2 and 57, respectively), with dashed black lines indicating the 95% confidence intervals. Spearman rank correlation coefficient ρ ( p-value) of -0.39 (<0.001).*

**Supplementary Figure 2B** Years Lived with Disability rate for iron

*All rates described are crude rates per 100,000 population. Grey vertical and horizontal gridlines indicate tertiles dividing lines for the measures: Years Lived with Disability (YLD) rates for iron deficiency (at 130 and 297) and cumulative of new ICU bed for COVID-19 (at 3.4 and 11.2).. Solid black line represents the restricted cubic spline, showing the shape of the adjusted association on a continuous scale with knots at the 5th, 35th, 65th and 95th percentiles (corresponding YLD rate for iron deficiency of 30, 135, 291 and 864, respectively), with black dashed lines indicating the 95% confidence intervals. Spearman rank correlation coefficient ρ ( p-value) of -0.43 (<0.001).*

**

**Supplementary Figure 2C** Years Lived with Disability rate for vitamin A deficiencies

*All rates described are crude rates per 100,000 population. Grey vertical and horizontal gridlines indicate tertiles dividing lines for the measures: Years Lived with Disability (YLD) rates for vitamin A deficiency (at 1.3 and 9.5) and cumulative of new ICU bed for COVID-19 (at 3.4 and 11.2).. Solid black line represents the restricted cubic spline, showing the shape of the adjusted association on a continuous scale with knots at the 5th, 35th, 65th and 95th percentiles (corresponding YLD rate for vitamin A deficiency of 0.1, 1.4, 9.2 and 80 respectively), with black dashed lines indicating the 95% confidence intervals. Spearman rank correlation coefficient ρ ( p-value) of -0.43 (<0.001).*

**Supplementary Figure 2D** Years Lived with Disability rate for high BMI

*All rates described are crude rates per 100,000 population. Grey vertical and horizontal gridlines indicate tertiles dividing lines for the measures: Years Lived with Disability (YLD) rates for high BMI (at 492 and 885) and cumulative of new ICU bed for COVID-19 (at 3.4 and 11.2). Solid black line represents the restricted cubic spline, showing the shape of the adjusted association on a continuous scale with knots at the 5th, 35th, 65th and 95th percentiles (corresponding to YLD rate for high BMI of 153, 510, 866 and 1398 respectively), and black dashed lines indicating the 95% confidence intervals. Spearman rank correlation coefficient ρ ( p-value) of 0.48 (<0.001).*

**Supplementary Figures 2** Scatterplots of countries’ vulnerability to malnutrition against the cumulative number of new beds for COVID-19 at the Intensive Care Unit until week 10 following the first confirmed death reported, stratified by income group [28].
